# Le cubitus varus post traumatique

**DOI:** 10.11604/pamj.2014.19.100.5322

**Published:** 2014-09-29

**Authors:** Karima Atarraf, My Abderrahmane Afifi

**Affiliations:** 1 Service d'Orthopédie Pédiatrique, CHU Hassan II, Faculté de Médecine et de Pharmacie, Université sidi Mohammed ben Abdullah, Fès, Maroc

**Keywords:** Cubitus varus, cal vicieux, supra condylienne, Cubitus varus, malunion, supracondylar

## Image en medicine

Le cubitus varus est la complication la plus fréquente des fractures supra condyliennes de l'enfant. Il est la conséquence d'un cal vicieux supra condylien survenu à la suite d'une réduction imparfaite ou d'un déplacement secondaire. En plus des conséquences fonctionnelles, le préjudice esthétique est très mal vécu par l'enfant et surtout par sa famille. C'est le cas de notre malade J. A. âgé de 13 ans, opéré à l’âge de 08 ans pour une fracture supra condylienne stade IV, traité par un embrochage en X après abord du foyer de fracture. L’évolution a été marquée par l'apparition d'une déformation du membre supérieur faite d'un varus de l'avant-bras par rapport au bras de 45° vu de face avec un déficit majeur de l'extension qui est compensée par la pronation, expliquant la photographie qui fait que la face postérieure du coude et de la main sont vus de face. Quand la déformation est importante une correction par ostéotomie de soustraction supra condylienne est indiquée, elle est réservée aux déviations supérieures à 15° de varus. Le meilleur traitement du cubitus varus reste sa prévention.

**Figure 1:**
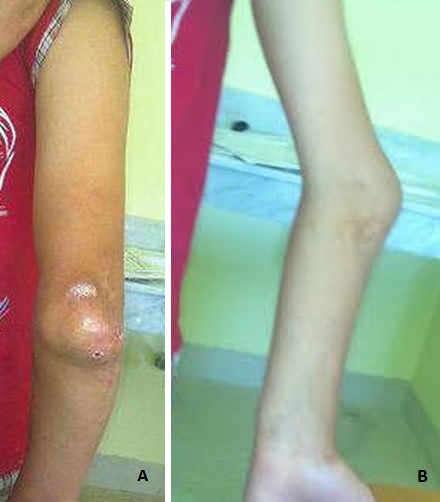
A) déficit de l'extension compensée par une pronation de l'avant bras qui fait que la face postérieure du coude est devenue antérieure; B) cubitus varus post traumatique de 45 gênant la fonction en plus de son aspect inesthétique

